# Medical and public health instructors’ perceptions of online teaching: A qualitative study using the Technology Acceptance Model 2

**DOI:** 10.1007/s10639-021-10681-2

**Published:** 2021-08-20

**Authors:** Meina Zhu, Yu Zhang

**Affiliations:** 1 Learning Design and Technology, Wayne State University, 5425 Gullen Mall, Detroit, MI 48202 USA; 2 Farmington Hills, MI USA

**Keywords:** Online teaching, Technology acceptance model, Medical and public health education, Instructor’s perceptions of online teaching

## Abstract

Many universities in the U.S. shifted from in-person teaching to online teaching due to the COVID-19 pandemic. Instructors’ acceptance of online teaching plays a crucial role, as the acceptance level can impact instructors’ online teaching behaviors. This qualitative study examined medicine and public health instructors’ perceptions of online teaching using the Technology Acceptance Model 2 (TAM2) model. Through semi-structured interviews with ten instructors in a Midwest university in the U.S., this study found that instructors had a high level of acceptance of online teaching. Instructors perceived the usefulness of online teaching in terms of learning objectives, assessment, instructional methods, and learning experience. Online teaching was perceived as useful overall, although challenges existed, such as online interaction, assessment, and hands-on practices. Regarding ease of use in online teaching, instructors perceived technology was easy to use; yet some pedagogical challenges existed, such as class engagement, the focus of learners’ attention, and transforming hands-on lab or clinical sessions online. The blended model is recommended to use for teaching and learning in medical and public health education post the pandemic. Detailed implications for practice and research were discussed in the end.

## Introduction

The COVID-19 pandemic has influenced the global education system from all settings (UNESCO, 2020), including U.S. higher education (Crawford et al., 2020). Harvard University announced to start moving courses online in March 2020 (Herpich, 2020). Later, many other universities in the U.S. also transitioned courses to online formats. Not surprisingly, under the circumstances, medical and public health education was also disrupted (Edigin et al., 2020; Ferrel & Ryan, 2020; Gaur et al., 2020; Rose, 2020). Many universities adopted recorded lectures or synchronous lectures to replace traditional face-to-face meetings. However, the shift to fully online learning during the pandemic (Liang et al., 2020) also led to challenges in medical and public health education (Franchi, 2020). The challenges of using online learning included unstable network infrastructure, such as hardware, software, network bandwidth, technical support, information literacy, online resources, etc. (Goh & Sandars, 2020; Liang et al., 2020; Rose, 2020). Besides, online education in the pandemic also caused pedagogical challenges to instructors, such as less social interaction and communication (Longhurst et al., 2020; Rose, 2020), developing online assessments as well as maintaining assessment integrity (Figueroa et al., 2020; Rose, 2020). Moreover, during transitioning courses online, the hands-on components such as face-to-face clerkship, clinical, and lab sessions were canceled (Gaur et al., 2020); and it made learners lose the opportunity to practice, collaborate, and build relationships (Edigin et al., 2020; Ferrel & Ryan, 2020; Gaur et al., 2020). Realizing the possible challenges that instructors in medical and health education may face up, this research attempts to delve into the challenges and support needed during online teaching from the perspectives of medical and health instructors. Through depicting the perceived advantages and drawbacks of online teaching practice, it revealed online teaching scenarios in medical and health education during the pandemic, indicated possible approaches to improve online teaching from pedagogical and technological perspectives during and post the pandemic, and suggested to use a blended model for teaching and learning in medical and public health education during and post the pandemic.

Also, this research aimed to explore instructors’ acceptance of online teaching in medical and public health education during the pandemic. The pandemic pushed the digital transformation of medical and health education. Under such circumstances, a line of research has provided theoretical guidance for online curriculum design and implementation, yet few studiesdelved into the instructors’ acceptance level during the transitional stage. In the context of forced course transition from in-person to online in medical and public health education during the pandemic, instructors’ acceptance of online education plays a crucial role, as the acceptance level can impact instructors’ online teaching behaviors. Prior studies (Gibson et al., 2008; Stewart et al., 2010) have explored faculty’s acceptance of online teaching before the pandemic. It was found that perceived usefulness strongly predicted faculty’s acceptance of online teaching, and perceived ease of use also predicted faculty’s acceptance of online teaching (Gibson et al., 2008). Since the pandemic has accelerated the online teaching transformation in medical and public health education, it is critical to examine instructors’ acceptance of online education at this time.

## Literature review

### Medical and public health education in the online teaching context

Online education has existed for decades. The rapid development of technology enables online education to be more effective and innovative (Dhawan, 2020). Online education provides flexible learning time and places (Dhawan, 2020; Singh & Thurman, 2019; Zhu et al., 2021). Students can customize learning processes based on their own needs. Instructors can combine different learning resources such as videos, audio, visual aids, and texts to engage learners in online education (Dhawan, 2020).

In the past decades, medical and public health education has transformed the pedagogy from teacher-centered and lecture-based to more student-centered, such as using problem-based learning, collaborative and self-directed learning (Buja, 2019; Singh et al., 2019), as well as using technology to enhance anatomy and lab sessions (Skochelak & Stack, 2017). Medical and public health education has increased more practices, laboratories, and clinical sessions for learners. Prior to the pandemic lockdown, some institutions used the blended model as an active learning approach in medical education (Buja, 2019).

In the context of the COVID-19 pandemic, many researchers (Scull et al., 2020; Sobaih et al., 2020; Stambouge et al., 2020; Torda, 2020) pinpointed that the demand for implementing online teaching in higher education had increased dramatically across the world. Various studies (Scull et al., 2020; Stambouge et al., 2020; Torda, 2020) acknowledged that the online teaching mode allowed learners to study flexibly in a remote setting, and therefore decreased the risk of contracting or spreading the Covid-19 virus. There is no exception for medical and public health education to adopt online teaching at this time. Meanwhile, transforming online teaching practice during the pandemic also led to innovation in medical and public health education, such as using virtual simulation and setting up fully online learning models. (Khalil et al., 2020).

### Theoretical framework

**Fig. 1:**
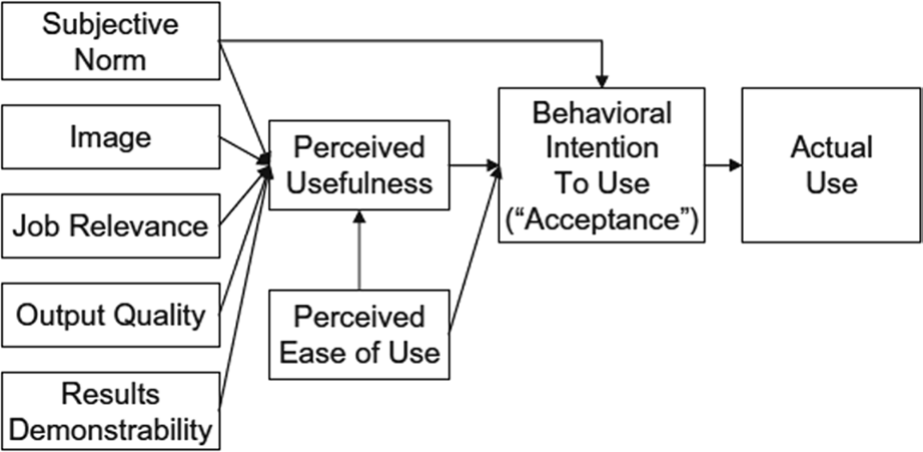
Technology acceptance model 2 from (Venkatesh & Davis, 2000)

The present study used the Technology Acceptance Model 2 (TAM2) (Venkatesh & Davis, 2000) as a theoretical framework (see Fig. 1). The Technology Acceptance Model (TAM) proposed by Davis (1989) examined users’ attitudes and behaviors towards adopting emerging technologies. In TAM, two primary factors that influence one’s acceptance of technology use are perceived usefulness (PU) and perceived ease of use (PEU). PU means that user's expectations regarding the degree that technology could enhance job performance (Davis, 1989). In an online learning context, PU is the extent that users perceive using online learning can improve teaching and learning outcomes (Sun & Zhang, 2008). PEU refers to the extent that individuals believe that using a specific technology is easy and straightforward (Davis, 1989). When a user perceives online learning as easy and takes less intense effort (Sun & Zhang, 2008), she/he will incline to continue using it. Abdel-Maksoud (2018) found that users’ PU and PEU were vital factors influencing whether users would accept a new technology. TAM2 is an extension of the TAM proposed by Davis (1989). As scholars found that PU is a strong predictor of technology acceptance, they thought it was vital to include the determinants of PU (Venkatesh & Davis, 2000). Therefore, TAM2 added the determinants of PU, such as subjective norm, image, job relevance, output quality, and results demonstrability. Subjective norms refer to users’ perceptions regarding whether they should adopt a technology system influenced by their peers within an organization (Venkatesh & Davis, 2000); job relevance is regarding user’s perceptions about how a technological system could help achieve goals in the job (Venkatesh & Davis, 2000); output quality means the required quality of technology that could be used for completing a specific task; result demonstrability is about the perceived benefits and demonstrable outcomes upon using a technological system (Venkatesh & Davis, 2000). Any component in PU may directly affect one’s behavioral intention to usetechnology. And the intention to use may influence the actual use.

The reason to choose the TAM2 in the present study is that it includes determinants that could possibly influence perceived usefulness. For the present research, instructors’ PU could be regarded as the degree to which instructor believes that teaching online with the use of technology can help boost teaching and learning performance. In terms of instructors’ PU of online teaching, we referred to the determinants of PU (Venkatesh & Davis, 2000) such as subjective norms, job relevance, output quality, and result demonstrability to help design initial interview guide questions.

### Perceived usefulness

PU is one primary determiner in predicting users’ behavioral attention to use new technology (Venkatesh & Davis, 2000). In the Information Systems field, a line of research followed Davis (1989)’s TAM model and showed PU could affect one’s behavior in using new technology (Abdullah et al., 2016; Chen et al., 2013; Elkaseh et al., 2016; Saade & Bahli, 2005).

Considering the critical role of PU in users’ technology acceptance, many researchers continued to delve into the determinants of PU of e-learning systems (Abdullah et al., 2016; Alsabawy et al., 2016; Shen et al., 2006; Teo, 2011; Venkatesh & Davis, 2000). Venkatesh and Davis (2000) found several constructs that influenced PU in TAM. These included the subjective norm, job relevance, output quality, and result demonstrability (Venkatesh & Davis, 2000). Teo (2011) found that several factors such as tutor attributes, course delivery as well as facilitating conditions were critical determinants of PU. Teo (2011) further suggested that these factors should be considered in the evaluation of the PU in the E-learning System.

As online teaching being used more frequently in the past three years, studies (Cherry & Flora, 2017; Mcgee et al., 2017; Wingo et al., 2017) examined faculty’s perceptions of online teaching in terms of their online teaching experience, teaching concerns as well as factors contributing to a positive online teaching attitude. For example, Wingo et al. (2017) synthesized various empirical studies regarding faculty’s online teaching experience through the lens of the TAM model. This conceptual review of the study (Wingo et al., 2017) revealed that faculty had concerns about students’ success in online teaching, course delivery mode, as well as their images as online instructors. Mcgee et al. (2017) focused on experienced online instructors’ experiences and beliefs during online teaching. The research (Mcgee et al., 2017) demonstrated expert online teachers’ skillset in online teaching and identified related institutional strategies that could support online instructors’ teaching delivery. Cherry and Flora (2017) conducted research regarding assessing radiography faculty’s perceptions towards the effectiveness of online teachings. In this research, Cherry and Flora (2017) analyzed factors that influenced faculty’s positive perceptions of effective online teaching, such as years of online teaching experience, the number of courses taught and perceived competence using technology.

### Perceived ease of use

PEU is critical for instructors to adopt online teaching. Research had found that instructors were dissatisfied with online teaching when they encountered technical barriers (Bolliger & Wasilik, 2009; Christianson et al., 2002) and struggled with learning how to use technology for online teaching (Kebritchi et al., 2017). When instructors found that online teaching increased their workload, they were also less satisfied (Bolliger & Wasilik, 2009; DeGagne & Walters, 2010).

Instructors tended to support online teaching more when they had more self-efficacy with technical skills (Zhen et al., 2008). Shea (2007) found that instructors’ technology skills were positively related to their willingness to teach online. Similarly, Osika et al. (2009) found that instructors’ prior success with technologies influenced their acceptance of using Learning Management Systems for online teaching.

## Research purpose and questions

The purpose of this study is to explore medicine and public health instructors' online teaching acceptance in higher education through using the Technology Acceptance Model 2 (TAM2). The TAM 2 is based on the TAM that emphasizes the perceptions of the potential technology user and further includes the determinants of perceived usefulness such as subjective norms, job relevance, output quality, and result demonstrability. This study examined instructor's perceived usefulness and perceived ease of use of online teaching, identified the challenges they encountered, as well as the support that instructors needed while shifting courses online. Three research questions guided this study are as follows:

1. How do instructors perceive the usefulness of online teaching?
2. How do instructors perceive the ease of teaching online?
3. How do instructors teach online?

## Methods

This study was conducted in a Midwest university in the U.S. The university is a public research university located in an urban area. The participants were instructors from the College of Pharmacy and Health Sciences and the College of Public Health. The inclusion of the participants was based on two criteria: (a) instructors who were teaching online in Fall 2020; b) instructors who were teaching undergraduate or graduate courses. This study adopted a qualitative exploratory interview study design (Maxwell, 1997). Maxwell (1997) emphasized a qualitative interview study as an interactive and exploratory process. The researchers conducted semi-structured interviews with ten participants through Zoom, a video conference tool (see Table 1). In semi-structured interviews, the interviewers do not necessarily need to follow a pre-formalized list of questions; instead, they ask more open-ended questions based on the flow of the conversation. Each interview, lasting approximately 30 minutes, was video-recorded and auto-transcribed through Zoom. The auto-transcripts were reviewed and revised by the researchers. The researchers conducted a member checking to ensure the accuracy of the transcripts. Among the ten interviewees, seven participants replied with confirmation or minor revisions with the transcripts. For example, one interviewee corrected the spelling of her transcript.

**Table 1:** Interviewees’ background information

Name	Occupation	Subject	Teaching Experience	Online Teaching Experience	Education	Course Level
Ben	Assistant Professor of Radiation Oncology	Medical Physics	5 years	Started teaching online during the pandemic	Ph.D.	Graduate courses & undergraduate courses
Cynthia	Clinical Instructor	Heath assessment, restorative care, and nursing practice	25 years	Only taught hybrid courses before	DNP	Undergraduate
Joyce	Clinical Instructor	Health assessment, nursing clinical practices	11 years	Started teaching online during the pandemic	DNP.; MS	Undergraduate
Carolyn	Associate Clinical Professor	Transcultural nursing, courses in physical experience	16 years	Started teaching hybrid during the pandemic	DNP	Undergraduate
Linda	Assistant professor	Oncology	5 years	Started teaching online during the pandemic	Ph.D.	Graduate
Cindy	Clinical Instructor	Acute care, nurse practitioner	4 years	Started teaching online during the pandemic	Ph.D.	Undergraduate
Megan	Assistant clinical professor	Clinical lab science	4 years	Completed hybrid class and 1–2 online courses before	Ed.D.	Undergraduate
Julia	Assistant Clinical professor	Clinical science in anesthesia	1.5 years	Completed online asynchronized session and synchronized session last fall	Ph.D.	Graduate
Laura	Assistant Clinical professor	Mortuary science	3 years	Many years in teaching online courses	Diverse education background	Undergraduate
Victoria	Program director and Assistant (Clinical) Professor	Pathologist assistance	6 years	Started teaching online during the pandemic	Diverse education background	Graduate

Notes: DNP-Doctoral of Nursing Practice.

The researchers analyzed data using thematic analysis (Braun & Clarke, 2006; Braun et al., 2014), a qualitative data analysis method that involves reading through data, such as interview transcripts, and then identifying common themes in meaning across the dataset. Two researchers conducted inductive open coding with three transcripts and identified initial themes separately. Then they discussed the discrepancies and reached a consensus with a set of initial code categories. Using the initial codes, two researchers coded all the transcripts individually and refined the themes. The researchers calculated the inter-rater reliability (0.93) by dividing the number of consent categories by the total categories. In this research, there are 40 categories in total and 37 are consent categories; the inter-rater reliability is 0.93. The final themes included three types of categories with eight sub-themes (Table 2).

**Table 2:** The final themes

Themes	Sub-themes
PU	Learning objectives
	Assessment
	Instructional methods
	Learning experience
PEU	Technology
	Pedagogy
Online teaching	Lecture
	Clinical and lab sessions

Notes: PU refers to perceived usefulness; PEU refers to perceived ease of use.

## Findings

In general, the ten interviewees reported that in the context of COVID-19 pandemic, online teaching was a useful approach to facilitate teaching and learning. Without the requirements of physically attending class, online teaching provided both students and instructors a safe, healthy, and less stressful teaching and learning environment.

### Instructors’ perceived usefulness of online teaching

Instructors’ perceptions towards the usefulness of online teaching were categorized into four dimensions, including learning objectives, instructional methods, assessment as well as learning experiences.

#### Learning objectives

Learning objectives state what students should know and be able to do following a period of instruction (Allan, 1996). Interviewees showed different perceptions towards learning objectives in transforming courses online. Whether maintaining learning objectives during online teaching depends on many factors, such as course credentials, course content, and online teaching modalities. Most of the interviewees stated that they kept the same learning objectives as in-person classes. Laura, in health education, stated:

If you look at my online classes and my in-person classes in Canvas, you would see that they look almost identical. The activities will change slightly because the online modality requires a little bit of different methods of delivery. But in terms of my objectives and the way I lay out the entire week, they are almost identical.

Regarding practical-based components, the level of difficulty in realizing these learning objectives was differentiated. The interviewees reported the easiness of fulfilling learning objectives in some online lab sessions, including running programs on computers and conducting clinical experience in patient care. Ben said: “I don't think online education is hampering their ability to learn the core tenants of the course.”

Also, for lab sessions including patient care in health education, interviewees stated that conducting patient care activities online was also easy. Students were invited to play the role as nurses online; by doing this, they were familiar with the process of disease diagnosis and prescription. Cindy stated:

There are several different technologies that I used to do these virtual simulations. One of them is a program, like a Canadian Vista, which stands for virtual simulation. And the Canadian Vista was like a video of patient interaction with a nurse and a patient. Along with the video, while you're watching it, it has questions embedded into it. And then, in the end, it'll give them a certificate of completion and grade them for a pass or fail.

Yet, several interviewees reported they found it challenging to conduct hands-on experiments online in health and public health education. For example, Victoria mentioned, “a lot of our objectives require psychomotor learning. For our clinical practice involves the outpatient understanding of texture, description of what grossly with the naked eye was able to see, and that doesn't be transited into a virtual world.”

Interviewees noted that they still needed to hold in-person clinical sessions. If onsite clinical sessions were not applicable due to the pandemic, hands-on components listed in the course outcomes would have to be suspended. Victoria said: “the psychomotor skills that we were unable to meet online, we had to postpone the labs until the time we were allowed to be back into the building to meet those objectives.”

#### Instructional methods

Instructors reported that instructional strategies should be adjusted during online teaching. In participants' views, effective instructional delivery required instructors' ongoing interactions with students. Yet in online teaching, they were not able to monitor students' first responses and practices in learning new things, as Ben stated:

Because they're watching the recording, they can't ask any questions, so it's harder to tailor the lecture to what students need... It's harder to gauge what they're thinking, and I usually look at their faces and see how they're reacting to things, asking questions, seeing what they do, and being there anymore, so it's hard to do so.

Participants mentioned that they needed to make online teaching more interactive, such as making more interactive live lectures and inserting question and answer sessions into pre-recorded lectures. Megan said, "I tried to create interactive lectures that are recorded so I can stop and have them answer questions during the pre-recorded lesson for marks." In addition, interviewees suggested effective online teaching strategies such as establishing a more structured online teaching and learning setting, providing initial posted questions ahead of class time, using mediated communication, and periodic checks.

#### Assessment

Researchers viewed assessment as a core component for effective learning (Bransford et al., 2000). In this research, all the interviewees considered assessment important in assessing students' learning performances. Instructors had concerns about academic integrity in online assessment. Some interviewees mentioned they had lost control in supervising students during the online test. Instructors claimed that students' overall performance in online tests tended to be higher than taking tests onsite. They doubted that if the students' scores online could accurately reflect what they have learned. Carolyn explained: “I have more concerns about students' ability to game the system, and their exam scores may not really reflect if they were sitting in a classroom, having to take that same exam.”

To address the issues of online test cheating, instructors suggested the use of an open-book test design. Yet challenges occurred during the test design process, such as developing a high-quality open-book test. Ben indicated, “I think the best way to avoid them cheating with an online exam would be to give them an open-book test, to give them a test that encourages the use of the notes.”

In addition, regarding assessing students' performance in online lab sessions, instructors found it challenging to evaluate students' practical skills, as they had limited access to work with students in the field of real-world settings. However, they used tests to check students' cognitive understanding of lab sessions. For example, Victoria stated: “they were assessed on their cognitive understanding of what they're supposed to be doing in the lab setting. The cognitive aspect was all meant.”

#### Learning experience

Overall, all the interviewees agreed that online learning was flexible for students. With all the available resources, students could be more prepared for class and review the lessons based on their learning needs. For example, several instructors mentioned the convenience of using pre-recorded video clips for lecture parts. In their views, students could pre-watch and re-watch the uploaded videos online anytime and anywhere as needed. Ben shared his experience in the following: “having the recorded one will be helpful because you could go back and slow down the speech and listen to my answers, which students have been thankful for.”

To enhance the learning experience during the pandemic, interviewees regarded the current teaching model should be a combination of synchronized and asynchronized sessions. A synchronized session allows students to interact with instructors and classmates and clarify specific knowledge points through live virtual instructions, and an asynchronized session guides students to pre-review and review the lessons. As Victoria suggested: "A type of modified classroom, the flipped classroom methodology, going forward, where I would have, you know, all my lectures already being recorded and, you know, we could facilitate a synchronous platform with the students."

Besides, the interviewees perceived that many factors, such as accessibility of technology tools, using learning resources, instructors' teaching styles, and learners' attributes, impacted online learning success. One factor that influenced the online learning experience was the accessibility of technology tools and learning resources. Most of the instructors expressed that they had tried to make all learning resources available online, yet it was difficult for students to utilize them. For example, Laura mentioned:

We also make assumptions that every student has access to technology, and they don't. A lot of students are trying to complete courses on their phones, a lot of these different tools and modalities that we have to use. They are very high-end data usage. It's very difficult for the students to be able to utilize all of the things that we make available to them.

Other factors, including teaching styles and learners’ characteristics, also affected the online learning experience. Several interviewees expressed that the instructor's ability to use online tools affected students' online learning satisfaction. An online course with a clear organization was effective from Cindy's perspective: “I had planned everything out with my 86 students. I think it was very well organized, and I think it can be very effective.” Moreover, many interviewees believed that the more self-disciplined and self-directed the students were, the more they would gain from online learning. In contrast, students who usually lagged behind others in traditional learning would also have issues during online learning.

### Instructors’ perceived ease of use during online teaching

Regarding the ease of use during online teaching, the medicine and public health instructors reported that their perceptions from two aspects: technology and pedagogy. In general, the ten interviewees reported that technologies were easy to use, yet some pedagogical challenges existed, such as class engagement, the focus of learners’ attention, and transforming hands-on lab or clinical sessions online during online teaching.

#### Technological perspective

The interviewees stated that they encountered minor challenges of using technology tools in online teaching at the beginning. However, the challenges were addressed quickly. A majority of them reported that technology tools such as Zoom and Big Blue Button were intuitive and easy to use. For instance, Cynthia stated that:

Initially, it was challenging because, again, I wasn't familiar with these different technologies. It was like just Friday, we were in class, and Monday, we were on Big Blue Button. And I had never used it before, but it was intuitive as you went through it… It became fairly easy [to learn] on my own.

Despite that the technology tools were easy for the instructors to operate, challenges that were out of control existed, such as students' technology and Wi-Fi stability. First, the Internet stability from both sides of the instructor and students during online teaching was not guaranteed. The unstable Internet may seriously interfere with the flow of instruction. For example, Julia said, "we've noticed a lot of times like their computers will cut out or they'll be texting us like ‘I can't hear the lecture,’ ‘I can't see this or the computers freezing.’ ".

Besides, online teaching encountered classroom management issues. When instructors held live synchronous sessions and shared the screen during the presentation, they could not see students' chat window on Zoom. If students asked questions during the presentation, the instructors could not notice and address their questions. Some instructors said that they might need a teaching assistant to help manage the chat window. For example, Ben described his situation, “it's hard to teach and watch the chatbox as you teach on Zoom. I've done small things in the community where I had like a technical assistant who was on Zoom watching the chat.”

#### Pedagogical perspective

Regarding the pedagogical perspective, the interviewees reported the ease of use of online teaching from the perspectives of engagement, lab, and clinical sessions.

Engaging learners is important in both face-to-face and online teaching. The instructors reported that it was more challenging to engage learners in online teaching. For example, Julia stated, "here are some learners who just sit there on Zoom and not say anything… I see a lot of that, or people aren't engaging." Carolyn shared her experience of engaging learners by letting them turn on cameras, "you always have to say, I want to see your faces. So that means you have to turn your camera on… I just think that it's harder to engage students when it's all online." While Linda suggested using a point value to engage learners: “I think with online classes, the way you have to engage students is to have a point value associated with it.”

Online learners also faced distractions at home. For instance, Julia said, "I think [students] they're so distracted at home, I also feel like when they're just a little picture on Zoom and not sitting there in the classroom: sometimes they think they cannot talk or participate as much."

Transferring laboratory sessions online was another challenge. Some lab sessions could be transferred to an online format. The instruments needed in lab sessions could be delivered to students' homes. For instance, Victoria shared how she handled lab sessions,

I purchased gift cards for Amazon for every one of my students. And with those gift cards, they were supposed to buy a baby doll, shipped to their home. We did the entire assignment that they would have done in the lab. We did it virtually as well.

Another way to transfer lab sessions was to use virtual reality tools or 3D models. However, it is difficult to find an appropriate 3D program to facilitate online teaching, and sometimes the tools were costly for the instructors and the department. Some lab sessions or clinical sessions could not even be able to transfer online. Megan said, " because we have a lot of hands-on [sessions] in our program, I feel it's going to be hard to do the lecture portion online and then combine that with the alive in person. So, I would like to keep those traditions in personal sessions."

Specifically, it is very challenging for the clinical session. Cindy said: "honestly, it was really like I said before, the clinical piece of it, is the biggest challenge that I had was trying to figure out, how am I going to, give them a good patient clinical experience without patients." Some instructors collaborated with a clinical preceptor in hospitals for clinical sessions.

From both pedagogical and technological perspectives, instructors reported that it was very time-consuming. As Linda said, "I find online teaching if you're going to do it well, very time-consuming. It takes a lot of effort to do it well. Designing a course takes a lot of time."

### Strategies of teaching online

According to the interviewees, online teaching mainly included lecture sessions and clinical/lab sessions. Lecture sessions usually were didactic, where instructors delivered content knowledge, further explained vital points, and clarified certain concepts to students. The lectures were in two formats: synchronous sessions and asynchronous sessions. In synchronous sessions, instructors used the following approaches to facilitate learning: 1) live lectures with recordings; 2) live lectures with discussions; 3) live office hours; 4) online labs; 5) flipped online learning. In asynchronous sessions, instructors utilized various methods to support students' learning: 1) providing recorded lectures that cover the core content; 2) using online discussion forums to answer students' questions, monitor students' learning, and engage students; 3) providing mini videos with embedded assessments to check students' understandings; 4) delivering an online survey to follow up with students' learning needs and learning progress; 5) providing students' timely ongoing feedback through learning management system. Clinical/lab sessions are integral parts of the curriculum in medical and public health education. Due to the impact of the pandemic, instructors also transferred parts of clinical sessions online. Clinical/lab sessions were in both synchronous and asynchronous formats. In general, clinical/lab sessions were conducted with various approaches, such as using virtual settings and simulations, working at home-based virtual labs, and collaborating with a clinical preceptor onsite. For instance, regarding using virtual environment and simulations, in one course, Ben made a demo in running a program on a computer, and then his students were required to work on a computer programming task as a practice. Regarding conducting home-based virtual lab sessions, for example, Victoria taught a course in forensic pathology and autopsy techniques, she first mailed tools to individual students, and then she did the real autopsy online by using a fetal specimen in the lab session. Following instructions from the instructor, students completed anatomy assignments at home using delivered baby dolls as substitutes for fetuses.

Regarding collaborating with a clinical preceptor onsite, interviewees in multiple courses stated that they had an additional onsite clinical preceptor who helped in supervising students' clinical sessions. Under this situation, course instructors were not in charge of clinical sessions; they only needed to collaborate with a clinical preceptor, such as facilitating onsite clinical experience and gathering clinical reports. Among the various teaching approaches in online lab sessions, virtual simulations in inpatient care and 3D models for video-based dissection anatomy were widely used by health and medical instructors. Instructors indicated that current options for virtual lab platforms were limited, and most online platforms were expensive to afford, so they chosefree trial platforms for online clinical sessions.

## Discussions

This study aimed to explore medicine and public health instructors' online teaching acceptance in higher education through examining instructor's PU and PEU during online teaching, as well as the challenges they have encountered. The study used the TAM2 (Venkatesh & Davis, 2000) as a theoretical framework and found that instructors had high acceptance of online teaching. To be specific, instructors perceived online teaching as useful. In addition, they perceived technology tools were easy to use, and challenges existed in some pedagogical aspects.

The findings of this study indicated that instructors encountered challenges of using technology at the initial stage ofonline course transition, then they learned the use of technologies through workshops and provided resources quickly. The findings differed from prior studies that technical barriers impeded online teaching (Bolliger & Wasilik, 2009; Christianson et al., 2002). One factor that contributes to the high level of instructors’ acceptance of online teaching in this study might be the influence of the external environment. Given that online teaching was the only approach to continue education during the pandemic, instructors had strong motivations to learn technology. However, instructors faced technology challenges that were out of their control, such as the instability of the Internet. This aligned with the prior researchers’ findings (Goh & Sandars, 2020; Liang et al., 2020; Rose, 2020). The technology issues could be solved when the hardware situation is being improved in society.

The findings of the study in terms of the instructors’ perceived usefulness of online teaching were aligned with the major constructs that influence PU in TAM2 (Venkatesh & Davis, 2000). The external environment, which forced the shifts to online teaching (Scull et al., 2020; Sobaih et al., 2020; Stambouge et al., 2020; Torda, 2020), created the new subjective norms of using online teaching and increased instructors’ perceived usefulness of online teaching. This finding confirmed that subjective norms influence PU in the TAM2 (Venkatesh & Davis, 2000). In addition, instructors in this study perceived that online teaching fulfilled its purpose in educating students during the pandemic and enabled students to achieve most of the learning objectives. This finding concurred with the finding of Wingo et al. (2017) that instructors valued students’ success inonline learning environment.

Although instructors perceived online teaching as useful in general, they also reported that it was not easy to use online teaching to realize some pedagogical purposes. Evidence from the research showed that fostering vibrant interactions with students during online teaching was a major challenge for instructors. This finding concurred with recent research on teaching online in medical education, such as Longhurst et al. (2020) and Rose (2020). To address this challenge, the following approaches were suggested. First, proficiency in using different technology tools and accompanied functions such as Zoom, Canvas, email is a prerequisite for instructors to facilitate online interactions. Second, maintaining a normal online meeting routinely with students is necessary during online teaching, such as setting up regular online conferences and holding office hours. During online meetings, instructors could provide explicit directions and requirements for online participation, clarify certain knowledge points, address students’ questions and concerns about the course, and follow up with students’ learning progress. Even interactions occur remotely, it still helps to create a kind of supportive online learning environment. Third, it would be useful to incorporate and design meaningful learning activities in fostering online interactions. For example, while addressing conceptual knowledge during an online lecture, instructors could check students’ understanding of the concept and invite students’ participation in using real-life examples and applications. If needed, student group work and projects could be assigned to promote student social and academic interactions in class (Masrom et al., 2021). In addition, it would be helpful to enhance the quality of instruction and communication with students online when an instructor encourages students to turn on their cameras during a synchronous meeting or use learning analytics to monitor medical students’ learning progress (Cirigliano et al., 2020).

Besides, instructors in this study perceived that some clerkship, clinical, and lab sessions could not be replaced by online teaching. Instructors emphasized the importance of having these hands-on sessions to let students practice and build relationships with peers and other professionals. Recent studies in medical education (Edigin et al., 2020; Ferrel & Ryan, 2020; Gaur et al., 2020) had similar findings. Therefore, although the online delivery mode could cover the knowledge content in medical and public health education, it was challenging for clerkship and clinical sessions. A blended model would be a good fit for future medical education after the pandemic.

This research found that instructors had concerns about online assessment. This finding is aligned with the finding from other studies (Aziz et al., 2020; Figueroa et al., 2020; Rose, 2020). Instructors in this research were mainly concerned about students cheating online and the quality design of online assessment. Based on the findings, students could cheat online by referring to online resources and texting answers with each other while taking online exams. To avoid students cheating online, the use of new technology is required during online exam proctoring. For example, a web camera could be used to monitor students’ behaviors; technology tools that help block the website and access to online learning resources could also be utilized for online exams. Technology-enhanced support during the online exam can reduce the chance of cheating and secure the quality assurance of exam outcomes. Meanwhile, instructors’ visibility in online proctoring is also important. The instructor should be responsible for supervising students during an online exam. They need to be conscious of their responsibilities during online exams proctoring, such as watching students online continuously, being mindful of students’ online cheating behaviors, and warning students who might break up the rules while taking online exams. In addition, credible technologies and additional supported staff need to be provided to help ensure the implementation of a reliable online exam upon instructors’ request. The quality design of online assessment was another concern reported by the instructors. The findings showed that instructors had challenges, especially in designing open-book tests and evaluating students’ practical skills. Although related studies (McCracken et al., 2012; Okada et al., 2019; Villiers et al., 2016) showed principles and strategies in designing online assessment, yet they had not fully provided detailed guidance in developing assessment on discipline-specific practices and approaches. Regarding designing high-quality online assessments, instructors should be encouraged to seek help and advice from their peers and organizations; more related trainings that target improving one’s skill set in test design could be offered, and more available resources in assessment within a specialization area are expected to be provided.

## Limitations

Several limitations existed in this study. First, the authors only included one data source in this study. If the researchers could review participants' courses and obtain perspectives from students' views to triangulate the data, it would enhance the trustworthiness of the study. Future researchers could obtain students' perceptions and experiences of effective online teaching strategies in the medical and public health field. Second, the data source was only from instructors from two colleges at one university. The generalization of the study findings to other universities or subjects should be cautious. Future research could expand the study in different universities.

## Significance of study and implications

This research has provided a snapshot of instructors’ perceived usefulness and perceived ease of use of online teaching in medical and public health education during the pandemic. From a theoretical perspective, the result of this study served as an indicator to reveal the medical and public health instructors’ current acceptance level of online teaching in the context of the COVID-19 environment using the TAM2. From a practical perspective, the related online teaching pedagogy reported by the instructors provided reference and pedagogical suggestions for educators in the field. For example, the teaching approaches such as inserting short questions into pre-recorded lectures and using breakout rooms to facilitate students’ interactions might be encouraged to use more widely in online teaching. The challenges demonstrated in this study also informed areas of improvement for online teaching in medical and public health education, such as the development of virtual technology platforms to support online teaching and the transformation of clinical sessions online. From a broader perspective, online teaching will likely be the ever-growing trend in education during the pandemic and even the post-pandemic era. This research could guide potential audiences, including policymakers in higher education, scholars, and faculty members, to rethink the new norms of teaching utilizing technology and reformation in medical and public health education in the context of the pandemic. More research could address the influence of this paradigm shift from the perspectives of policymakers, students, and other administrators in medical and public health education. For instance, what roles that institution plays in fostering online teaching; how to promote the implementation of new technology tools and online learning platforms for online teaching; what the students’ performance are and their acceptance levels in online education; and what the possible trends and issues are in future medical and public health education.

## Conclusions

In conclusion, medicine and public health instructors accepted online teaching during the pandemic. In this study, instructors recognized the usefulness of online teaching, and perceived technology of online teaching was easy to use. Yet, the challenges existed, especially in the areas of pedagogy and transformation of clinical and lab sessions. Informed by the findings, we concluded that online teaching in medical and public health education requires careful course design to meet course objectives, appropriate adjustment of instructional methods to the fitting of online teaching environment, and different levels of support to ensure successful implementation of online teaching, as well as the quality of assurance in assessing students’ learning outcomes. In addition, given that clinical and lab sessions were difficult to be transformed online completely, we suggested that using a blended learning model that combines online teaching and face-to-face clinical and lab sessions in medical and public health education during the pandemic and post the pandemic.

## Data Availability

The datasets used and/or analyzed during the current study are not publicly available due to their personal and private nature but are available from the corresponding author on reasonable request.
